# Integrative targeted blood gene-expression profiling, coding-region genetic variants, and machine learning identify candidate biomarkers of milk fever in Holstein dairy cattle

**DOI:** 10.3389/fvets.2026.1946092

**Published:** 2026-09-21

**Authors:** Basma Mohamed Hendam, Tahani M. I. Al-Hazani, Asmaa A. Badr, Khairiah Mubarak Alwutayd, Hanan M. Alharbi, Ashwag Shami, Mariam Abdulaziz Alkhateeb, Nawal Al-Hoshani, Ahmed M. El-Sebaey, Ahmed Ateya

**Affiliations:** 1 Department of Animal Wealth Development, Faculty of Veterinary Medicine, Mansoura University, Mansoura, Egypt; 2 Biology Department, College of Science and Humanities, Prince Sattam Bin Abdulaziz University, Al-Kharj, Saudi Arabia; 3 Department of Biology, College of Science, Princess Nourah bint Abdulrahman University, Riyadh, Saudi Arabia; 4 Department of Clinical Pathology, Faculty of Veterinary Medicine, Mansoura University, Mansoura, Egypt

**Keywords:** Holstein cattle, hypocalcemia, livestock genomics, machine learning, marker assisted selection, targeted blood gene-expression profiling

## Abstract

**Background:**

Milk fever is a serious metabolic disease affecting high-yielding dairy cows during the transition period and can result in substantial economic losses due to reduced milk production, impaired health, increased treatment costs, and culling.

**Methods:**

The present study aimed to identify molecular signatures associated with milk fever in Holstein dairy cows using targeted blood gene-expression profiling, genetic variation, and artificial neural network (ANN) modeling. One hundred multiparous Holstein cows were enrolled in the study, fifty of them clinically healthy and fifty diagnosed with milk fever based on clinical signs and serum calcium levels below 2.0 mmol/L. Quantitative real-time PCR was used to measure relative mRNA expression of 13 potential calcium signaling, endocrine control and cellular metabolism-related genes.

**Results:**

Single nucleotide polymorphisms (SNPs) were then detected by PCR product sequencing. ANN and hierarchical cluster analysis were used to assess the diagnostic performance of the biomarkers tested. Milk fever cows showed significant upregulation of *PKIB, ASIC2, PER1, NUAK1, STX1A, SRI* and *ITPR1*, and significant downregulation of *CAMK2A, ANXA6, CACNA1S, VDR, CAMK4* and *NESP55* (*p* < 0.05). Sequencing identified 23 SNPs in the coding region, of which 17 were synonymous and 6 non-synonymous. These variants showed significant differences in their distribution between healthy and affected cows (*p* < 0.001). The most informative biomarkers for the classification of the disease by ANN analysis were *NUAK1* and *NESP55*. Hierarchical clustering based on normalized gene-expression profiles also showed distinct clustering patterns between healthy and affected cows.

**Discussion:**

The identified gene-expression signatures and coding-region variants may provide candidate biomarkers and genetic markers for future studies aimed at reducing susceptibility to milk fever in dairy cattle. These results show that the combination of targeted blood gene-expression profiling, functional genetic variation, and machine-learning methods offers a powerful framework for genomic prediction, precision livestock management, and future marker-assisted breeding strategies.

## Introduction

Milk fever, also known as periparturient hypocalcemia, is a major metabolic disorder affecting high-yielding dairy cows during the transition period. It typically develops within the first 24–48 h after calving, when the sudden increase in calcium demand for colostrum and milk production exceeds the cow’s ability to maintain calcium homeostasis (1). Failure to restore adequate blood calcium levels results in clinical hypocalcemia, characterized by neuromuscular disturbances such as muscle weakness, recumbency, impaired circulation, and, in severe cases, death. Although many cows adapt successfully to the physiological demands of parturition and early lactation, some fail to maintain calcium homeostasis (2). Consequently, milk fever remains an important threat to dairy health and productivity, causing substantial economic losses through reduced milk production, impaired health, increased treatment costs, and culling.

There are profound endocrine and metabolic adaptations that accompany the onset of lactation in dairy cows. During early lactation, the rapid increase in milk production often exceeds the energy supplied through feed intake, resulting in a state of negative energy balance (NEB) (3, 4). To compensate for this energy deficit, cows mobilize body fat reserves to support milk synthesis, a physiological response that increases their susceptibility to metabolic disorders (5). Although many animals successfully adapt to this challenge without developing clinical disease, inadequate metabolic adaptation predisposes susceptible cows to several postpartum disorders, including ketosis, retained placenta, displaced abomasum, mastitis, metritis, and milk fever (6, 7). These conditions have substantial economic consequences for the dairy industry because they reduce milk yield, impair reproductive performance, increase veterinary costs, and contribute to premature culling (8).

Milk fever is usually diagnosed using clinical examination together with measurement of serum calcium concentration. Though these methods are highly reliable in detecting milk fever-affected animals, they are not helpful in knowing the changes that take place at the molecular level before the disease and also in understanding the biological reasons for variations in individual susceptibility. Therefore, more efforts have been made to find molecular markers which will help to predict the disease at an early stage (9).

Functional genomic research has enabled significant progress in elucidating the nature of complex diseases in animals. Blood transcriptome analysis has become an important method as blood leukocytes represent the systemic response to metabolism, hormones, and the immune system (9, 10). Transcriptomic research has led to the discovery of possible biomarkers related to several diseases in cattle, such as mastitis, ketosis, lameness, brisket disease, and growth retardation (9, 11–13). Targeted gene-expression biomarkers can provide information about disease-related biological processes in an earlier stage than conventional biochemical markers (14).

Recent advances in genomics in livestock have even contributed to the identification of functional genetic markers in relation to economically significant diseases (13). The use of high-throughput transcriptomics in combination with the candidate gene approach has helped in identifying molecular markers in relation to disease resistance/susceptibility and productivity in dairy cattle (12). Combining gene-expression patterns with functional DNA polymorphisms not only improves understanding of disease-related molecular mechanisms but also facilitates the identification of candidate genomic markers.

Genetic variation is another factor contributing to the risk of developing milk fever. Heritability of periparturient hypocalcemia has been estimated in previous research to be 0.01 to 0.35, depending on the particular population of animals and the analysis technique used (15). These estimates vary according to the population, definition of the hypocalcemia phenotype, and analytical approach used. Previous studies have considered phenotypes based on clinical diagnosis, serum calcium measurements, and related metabolic-disease indicators. Such variation highlights the complexity of defining and predicting periparturient hypocalcemia and supports the investigation of molecular biomarkers that may complement conventional clinical and biochemical measurements (15–17). In addition, Holstein cows suffer from milk fever more frequently than most other dairy cattle breeds, which indicates the contribution of the genetic factor in the disease onset (16). Thus, the identification of SNPs in genes responsible for calcium balance and metabolism can be beneficial for the identification of disease-related genetic markers for preventive purposes. Recently conducted genomics studies proved the existence of molecular differences between healthy and hypocalcemic cows (17).

As the field of molecular biology progresses, machine learning algorithms are now being used in the field of veterinary medicine to diagnose and predict diseases. Of all the techniques in machine learning, the most promising one that has emerged is the artificial neural network, which has been useful in modeling nonlinear associations in high-dimensional biological data sets. Using both the information from the transcriptome and genome, the artificial neural networks (ANNs) model are better able to classify diseases and biomarkers than using biomarkers alone (16).

Although there is an increase in the body of literature that supports the importance of the above techniques in the investigation of animal disorders, few attempts have been made in the integration of these complementary techniques in relation to milk fever. An analysis based on a combination of the blood transcriptional signature along with the identification of functional genetic variants will help shed light on the molecular bases of hypocalcemia and allow for the generation of better tools for diagnosis and prognosis. Thus, in the current study, we explored the expression levels of specific blood-derived genes associated with calcium regulation, endocrine control, and cellular metabolism. Moreover, DNA sequencing was done to identify the functional SNPs in these genes. Artificial neural networks and cluster analysis were done in relation to milk fever in Holstein dairy cows (18).

We hypothesized that integrating targeted gene-expression biomarkers, functional genetic polymorphisms, and artificial intelligence–based analytical approaches would improve the identification of biologically meaningful markers associated with milk fever susceptibility. Such an integrated strategy may also provide promising genomic targets for selective breeding and contribute to the development of precision health management programs in dairy cattle.

## Materials and methods

### Ethical approval

All experimental procedures involving animals were approved by the Mansoura University Animal Care and Use Committee (MU-ACUC), Faculty of Veterinary Medicine, Mansoura University, Egypt (approval no. VM.R.25.08.234). Blood sampling and clinical examinations were performed by licensed veterinarians using standard veterinary practices to minimize animal stress and discomfort.

### Clinical examination, research design, animals, and collection of blood samples

This study involved one hundred multiparous Holstein dairy cows (*Bos taurus*). The animals were kept in the same housing, nutrition, and management settings after being chosen from a commercial dairy farm in Egypt’s Ismailia Province. The cows were fed a total mixed feed prepared in accordance with lactation requirements and kept in a free-stall system with straw-bedded cubicles. Each cow in the herd produced about 8,500 kg of energy-corrected milk on average each year. Cows were divided into two groups (*n* = 50 each) based on serum calcium concentration and postpartum clinical examination: The 100 multiparous Holstein dairy cows were selected from the same commercial herd and maintained under identical housing, feeding, and routine management conditions. They comprised 50 healthy controls and 50 cows diagnosed with milk fever. Group assignment was based on postpartum clinical examination and serum total calcium concentration. The mean age was 4.3 ± 0.8 years in healthy cows and 4.4 ± 0.9 years in milk fever-affected cows, while the corresponding mean parity was 2.8 ± 0.9 and 2.9 ± 1.0, respectively. No significant differences were observed between the groups in age or parity (*p* > 0.05).

Cows were grouped as healthy and those with milk fever through both clinical assessment and serum total calcium concentration. The diagnosis was supported by a favorable response to intravenous calcium treatment. Healthy cows were those with normal serum total calcium concentration levels (between 2.0–2.6 mmol/L or 8.0–10.5 mg/dL) and lack of any clinical symptoms. Diagnosis of milk fever involved cows with clinical symptoms of hypocalcemia around the time of parturition alongside a serum total calcium concentration less than 2.0 mmol/L (8.0 mg/dL). These clinical symptoms included weakness of muscles, instability in their gait, inability to stand, lying down, coldness in their extremities, enlarged pupils, reduced consciousness, and low reactivity.

Each cow’s jugular vein was used to aseptically draw five milliliters of blood, which were then placed in sterile tubes with EDTA acting as an anticoagulant. Samples were processed right away for RNA extraction after being transported on ice.

### Isolation of RNA, evaluation of quality, synthesis of cDNA, and real-time quantitative PCR

Following the manufacturer’s recommendations, total RNA was extracted from whole blood using TRIzol reagent, and then purified using the RNeasy Mini Kit (Qiagen, Germany; Cat. No. 74104). A NanoDrop ND-1000 spectrophotometer (Thermo Scientific, USA) was used to assess the concentration and purity of RNA. For downstream analysis, only samples with a sufficient purity (A260/A280 ratio between 1.8 and 2.0) were utilized. The Revert Aid First Strand cDNA Synthesis Kit (Thermo Fisher Scientific, USA; Cat. No. EP0441) was used to create complementary DNA (cDNA) from purified RNA in accordance with the manufacturer’s instructions. Prior to quantitative PCR analysis, the resultant cDNA was kept at −20 °C.

Quantitative real-time PCR (qRT-PCR) based on SYBR Green was used to analyze gene expression. *CAMK2A, ANXA6, CACNA1S, VDR, CAMK4, PKIB, ASIC2, PER1*, *NUAK1, NESP55, STX1A, SRI,* and *ITPR1* were among the genes examined. Based on published *Bos taurus* sequences found in GenBank, primers were created (Table 1). The internal reference gene for normalization was *GAPDH*. Every 25 μL response included: 2 × SYBR Green Master Mix, 12.5 μL (Bioline, UK), 0.5 μL of 10 pmol of each primer, 3 μL of cDNA template. Add water free of nucleases to the desired volume. In a real-time PCR thermal cycler, amplification was performed under the following circumstances: reverse transcription for 30 min at 50 °C, first denaturation for 10 min at 94 °C, after 40 cycles of 15 s at 94 °C, 1 min at the gene-specific annealing temperature, and 30 s at 72 °C. At the conclusion of amplification, a melting curve analysis was carried out to verify the PCR products’ specificity. The 2^^−ΔΔCt^ technique was used to calculate relative gene expression (19).

**Table 1:** Real-time PCR primers made of oligonucleotides that are forward and reverse for genes under study.

Investigated marker	Primer	Product size (bp)	Annealing temperature (°C)	GenBank isolate
*CAMK2A*	F5′- CCAGAAGTGCTGCGGAAGGACC -3 R5′- TCCTCAATGGTGGTATTGGTGC -3′	476	58	NM_001075938.1
*ANXA6*	F5′- ACCGATGAGAAGTGCCTCATTG -3 R5′- TCGGATGCTGGCTTCAATCGGC -3′	366	60	NM_001103224.1
*CACNA1S*	F5′- ACTGAAATTGAGATGGAGGAGAT -3′ R5′- CAAGGCCCTCTCTGTTCATCC -3′	438	60	XM_059875843.1
*VDR*	F5′- CACAAGACCTACGACGACACCT -3′ R5′- CGCTGACTTGGTACTTGTAGTC -3′	472	60	NM_001167932.2
*CAMK4*	F5′- GTAGAACATCAAGTACTCATGA -3′ R5′- TCCTGGACGCCGCTGTGGCTGC -3′	479	58	ON961763.1
*PKIB*	F5′- ATGAGGACAGATTCACCCAAAAT -3′ R5′- TCCTTCATCTTTGGGCTTTTCC -3′	234	58	NM_001114518.2
*ASIC2*	F5′- ATCATGCTGGACATTCAGCAGGA -3′ R5′- CAGGCTCTGCACACTCCTTGTG -3′	382	58	NM_001076484.1
*PER1*	F5′- GAGCAGGCCTTCCTCAGCCGCT -3′ R5′- GAACAGATAGTTAGGGAGCACCA -3′	414	60	XM_059878047.1
*NUAK1*	F5′- GTGCTGGAGCGTCAGCGGTCG -3′ R5′- GATGAATAGTAACCCGATTCTC -3′	362	58	NM_001205496.1
*NESP55*	F5′- GATCGTAGATCCCGACCTCAG -3′ R5′- GCTCCGCAGCCTCAGGGCGCTG -3′	495	60	U77614.1
*STX1A*	F5′- ATCGAGCAGAGCATCGAGCAGGA -3′ R5′- CGGCCTTCTTGGTGTCAGACACG -3′	478	58	NM_001083798.1
*SRI*	F5′- ACTCAGGATCCGCTGTATGGTTA -3′ R5′- CACTCATGACACACTGGATGA -3′	502	58	NM_001427871.1
*ITPR1*	F5′- TTAGCAGCAGAGGTAGACCCTG -3′ R5′- CTCAGCAGGAGAGACTGGCACTA -3′	378	60	NM_001435139.1
*GAPDH*	F5′- AGGTCGGAGTGAACGGATTC -3′ R5′- GATGTTGGCAGGATCTCGCT -3′	242	58	NM_001034034.2

CAMK2A, Calcium/calmodulin dependent protein kinase II alpha; ANXA6, Annexin A6; CACNA1S, Calcium voltage-gated channel subunit alpha1 S; VDR, Vitamin D receptor; CAMK4, Calcium/calmodulin dependent protein kinase IV; PKIB, Protein kinase inhibitor beta; ASIC2, Acid sensing ion channel subunit 2; PER1, Period circadian regulator 1; NUAK1, NUAK family kinase 1; NESP55, Neuroendocrine secretory protein 55; STX1A, Syntaxin 1A; SRI, Sorcin; ITPR1, Inositol 1,4,5-trisphosphate receptor type 1.

### DNA sequencing, PCR amplification, and SNP detection

Following the manufacturer’s instructions, amplified qPCR products of the anticipated size were purified using a PCR purification kit (Jena Bioscience, Germany; Cat. No. pp-201×s/Germany). A UV–Vis spectrophotometer (Q5000, USA) was used to quantify the purified products.

An ABI 3730XL DNA sequencer (Applied Biosystems, USA) based on the Sanger chain-termination method (20) was used for bidirectional sequencing. Chromas 1.45 was used to examine sequence chromatograms, and BLAST 2.0 was used to align nucleotides (21). By comparing the appropriate reference sequences that were obtained from GenBank, single nucleotide polymorphisms (SNPs) were found. MEGA6 software was used to further analyze sequence alignments and amino acid changes (22).

### Artificial neural network and hierarchical cluster analysis

An artificial neural network (ANN) analysis was performed using SPSS Statistics version 29 to evaluate the discriminatory capacity of the targeted gene-expression biomarkers between healthy and milk fever-affected cows. The ANN model was developed using the 100 cows included in the study, with the expression levels of the 13 candidate genes used as input variables and clinical status (healthy versus milk fever) as the output variable. The network architecture, training algorithm, activation functions, data partitioning, stopping criteria, and model-evaluation procedure were recorded and are reported to facilitate reproducibility. The performance of the ANN was evaluated using accuracy, sensitivity, specificity, F1 score, and the area under the receiver operating characteristic curve (AUC). The ANN achieved an accuracy of 98%, sensitivity of 97%, specificity of 99%, F1 score of 0.98, and AUC of 0.98.

Hierarchical cluster analysis was performed using normalized gene expression data to evaluate similarities among gene-expression profiles and to visualize clustering patterns between healthy and affected cows. Dendrograms were generated based on Euclidean distance and average linkage methods.

### Statistical analysis

SPSS software version 23 (IBM Corp., USA) was used to conduct statistical analyses. Mean ± standard error (SE) was used to express the data. The independent samples *t*-test was used to assess differences in gene expression between milk fever and healthy cows. The Chi-square test was used to examine the distribution of SNP frequencies among the groups. The ability of the found genetic markers to discriminate cows based on their health condition was evaluated using a linear discriminant analysis (LDA). *p* < 0.05 was used to determine statistical significance.

Prior to LDA, the suitability of the data for discriminant analysis was assessed based on the assumptions of multivariate normality of the predictor variables and homogeneity of covariance matrices across groups. The LDA model was used to classify cows according to health status based on the investigated genetic markers, and classification accuracy was evaluated from the resulting group membership matrix.

## Results

### Clinical findings

The diagnosis of milk fever in the afflicted Holstein dairy cows was validated by clinical and biochemical results. Muscle weakness, decreased ruminal motility, sternal or lateral recumbency, decreased responsiveness, and decreased feed intake were among the typical clinical signs displayed by milk fever-affected cows. Hypocalcemia was confirmed by the significantly lower serum calcium values in affected cows compared to clinically healthy controls (*p* < 0.05). The control group did not exhibit any clinical problems.

### Variations in candidate gene expression

Significant changes in the expression of genes related to cellular metabolism, endocrine control, and calcium signaling were shown by quantitative real-time PCR analysis (Figure 1). *PKIB, ASIC2, PER1, NUAK1, STX1A, SRI*, and *ITPR1* expression levels were considerably higher (*p* < 0.05) than those of healthy cows. On the other hand, cows with milk fever had significantly lower levels of *CAMK2A, ANXA6, CACNA1S, VDR*, *CAMK4,* and *NESP55* (*p* < 0.05). *NUAK1* was the upregulated gene with the biggest increase in transcript abundance, while *CACNA1S* was the downregulated gene with the most noticeable decrease.

**Figure 1:**
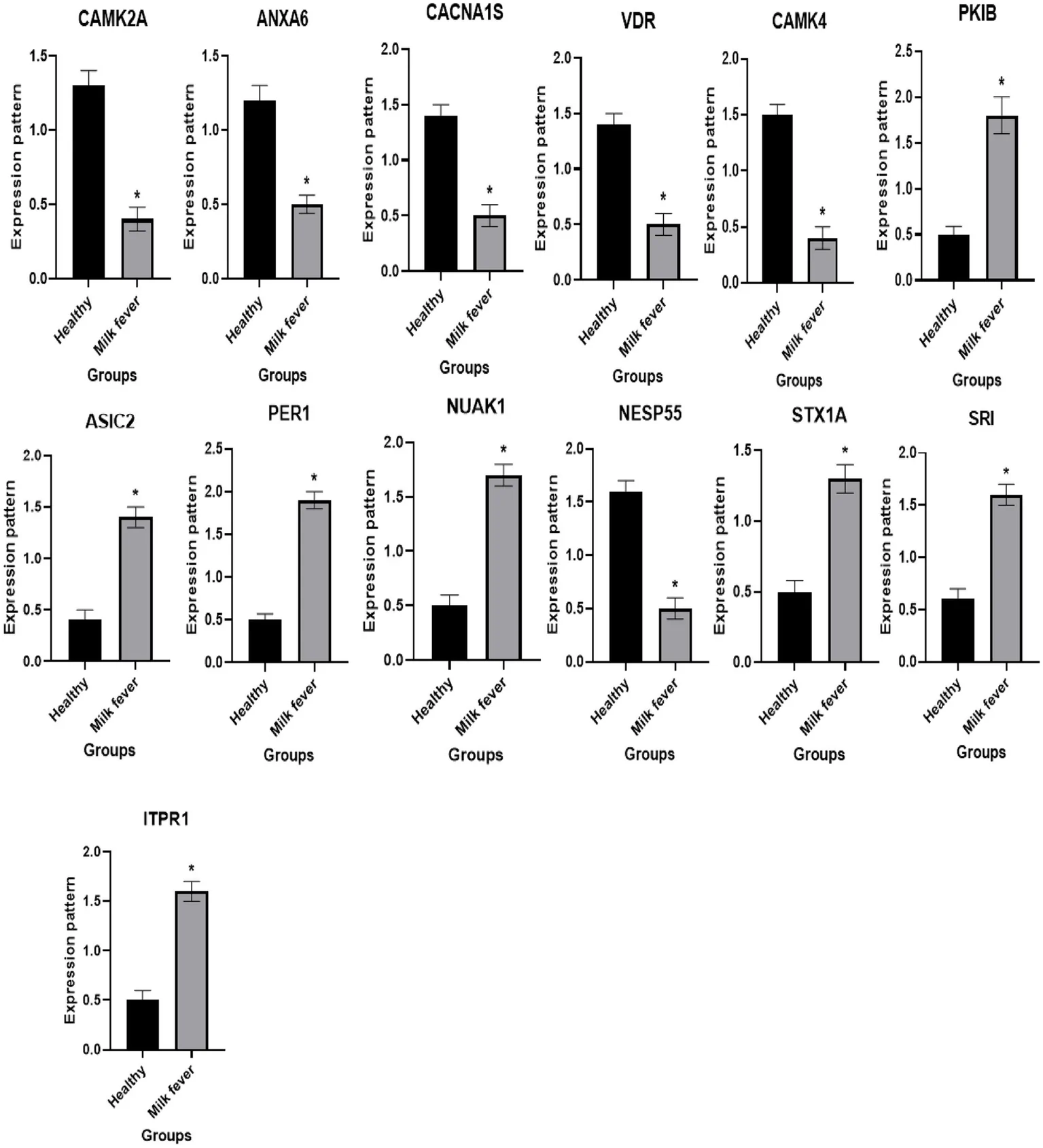
Different genes transcript levels between healthy and milk fever Holstein dairy cows. The symbol * denotes significance when *p* < 0.05.

### Finding genetic variants

Single amplicons of the anticipated sizes were produced by PCR amplification of the thirteen candidate genes. Twenty-three coding-region single nucleotide polymorphisms (SNPs) were found throughout the examined genes by direct sequencing of the purified PCR products. Of the variants found, six were nonsynonymous replacements that changed amino acids, and seventeen were synonymous alterations (Table 2). The majority of SNPs were found in conserved coding areas and were effectively matched with GenBank-deposited homologous *Bos taurus* reference sequences (Supplementary Figures S1–S13).

**Table 2:** Dispersion of investigated markers in milk fever affected and healthy Holstein cows with a single base differential and a potential genetic alteration.

Gene	SNPs	Healthy *n* = 50	Milk fever *n* = 50	Total *n* = 100	Chi square value X2	*p* value	Kind of inherited change	Amino acid order and sort
*CAMK2A*	G174C	26/50	-/50	26/100	35.1	0.001	Synonymous	58 T
	G183A	14/50	-/50	14/100	16.2	0.001	Synonymous	61 P
*ANXA6*	C216T	-/50	19/50	19/100	23.4	0.001	Synonymous	72 V
*CACNA1S*	C48T	31/50	-/50	31/100	44.9	0.001	Synonymous	16 D
	C99T	-/50	23/50	23/100	29.8	0.001	Synonymous	33 N
	C187G	-/50	16/50	16/100	19	0.001	Non-synonymous	63 P to A
	G386A	-/50	28/50	28/100	38.8	0.001	Non-synonymous	129 G to D
*VDR*	G193A	18/50	-/50	18/100	21.9	0.001	Synonymous	65 N
	T285C	29/50	-/50	29/100	40.8	0.001	Synonymous	95 Y
	T396G	-/50	34/50	34/100	51.5	0.001	Synonymous	132 L
*CAMK4*	G111A	24/50	-/50	24/100	31.5	0.001	Synonymous	37 G
*PKIB*	G102A	-/50	19/50	19/100	23.4	0.001	Synonymous	34 Q
*ASIC2*	T159C	-/50	33/50	33/100	49.2	0.001	Synonymous	53 F
	T294C	29/50	-/50	29/100	40.8	0.001	Synonymous	98 I
*PER1*	C93A	15/50	-/50	15/100	17.6	0.001	Synonymous	31 G
	G265A	27/50	-/50	27/100	36.9	0.001	Non-synonymous	89 A to T
	T313C	14/50	-/50	14/100	16.2	0.001	Non-synonymous	105 S to P
*NUAK1*	T269C	-/50	26/50	26/100	35.1	0.001	Non-synonymous	90 V to A
*NESP55*	T428C	-/50	36/50	36/100	56.2	0.001	Non-synonymous	143 V to A
*STX1A*	C444T	23/50	-/50	23/100	29.8	0.001	Synonymous	148 Y
*SRI*	C399T	17/50	-/50	17/100	20.4	0.001	Synonymous	133 C
	A462G	36/50	-/50	36/100	96	0.001	Synonymous	154 V
*ITPR1*	G183A	-/50	19/50	19/100	23.4	0.001	Synonymous	61 T

CAMK2A, Calcium/calmodulin dependent protein kinase II alpha; ANXA6, Annexin A6; CACNA1S, Calcium voltage-gated channel subunit alpha1 S; VDR, Vitamin D receptor; CAMK4, Calcium/calmodulin dependent protein kinase IV; PKIB, Protein kinase inhibitor beta; ASIC2, Acid sensing ion channel subunit 2; PER1, Period circadian regulator 1; NUAK1, NUAK family kinase 1; NESP55, Neuroendocrine secretory protein 55; STX1A, Syntaxin 1A; SRI, Sorcin; and ITPR1, Inositol 1,4,5-trisphosphate receptor type 1. A = Alanine; C = Cysteine; D = Aspartic acid; F = Phenylalanine; G = Glycine; I = Isoleucine; L = Leucine; N = Asparagine; P = Proline; Q = Glutamine; S = Serine; T = Threonine; V = Valine; and Y = Tyrosine.

The distribution of SNPs in a number of the examined genes differed significantly between milk fever and healthy cows. For every gene examined, chi-square analysis showed significant differences in genotype frequencies between milk fever and healthy cows (*p* < 0.05). Figure 2 shows an alluvial plot showing the distribution of the markers under investigation in 50 Holstein dairy cows with milk fever and 50 healthy cows. Hierarchical clustering based on normalized expression profiles produced two distinct clusters corresponding largely to the healthy and affected cows, indicating that the combined expression patterns captured group-related molecular differences. The robustness of the chosen molecular indicators in differentiating clinically healthy animals from affected cows was further supported by the clustering pattern.

**Figure 2:**
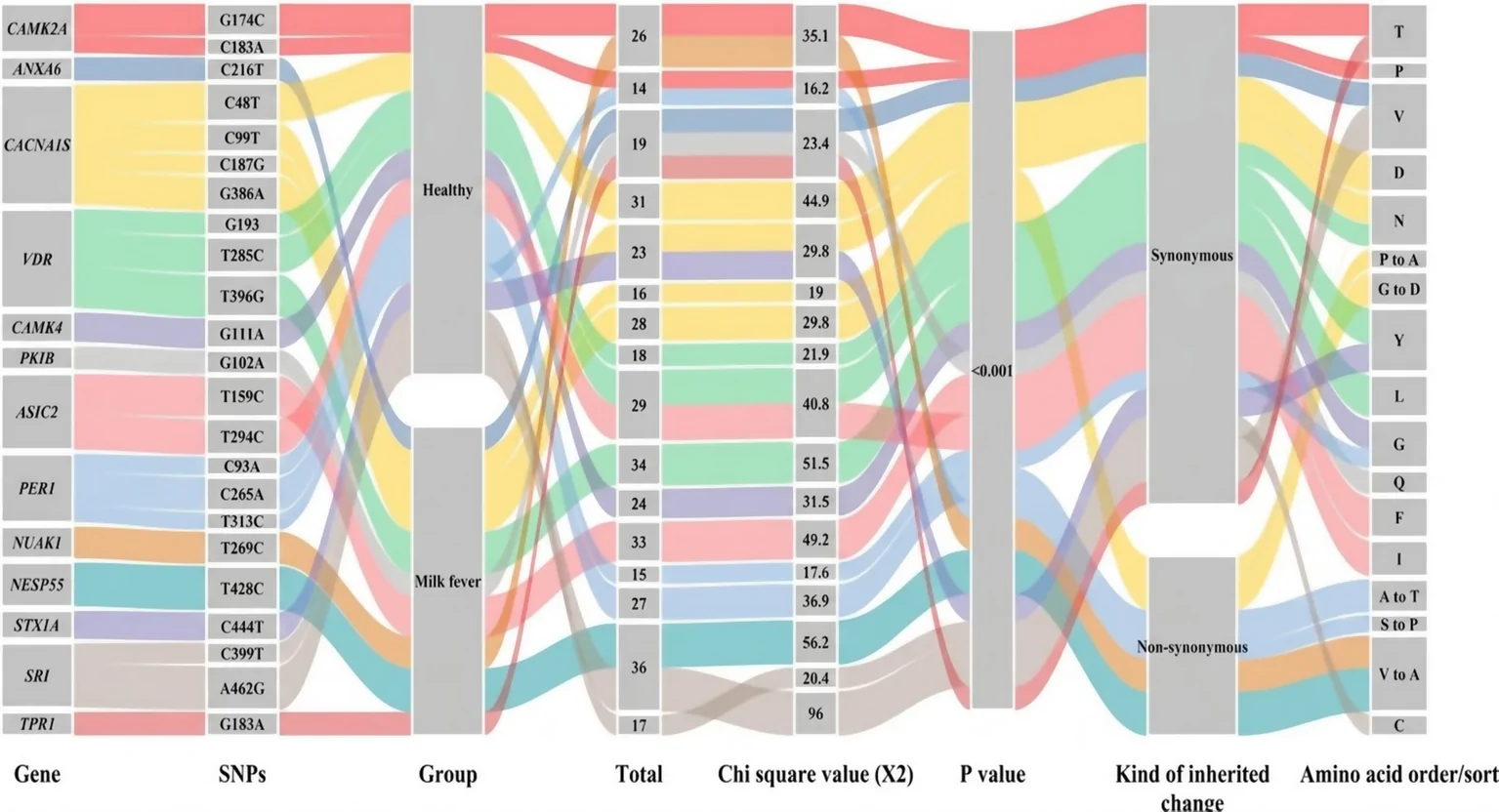
Alluvial plot showing the distribution of the identified coding-region SNPs among healthy (*n* = 50) and milk fever-affected (*n* = 50) Holstein dairy cows. The plot illustrates the relationship among genes, identified SNPs, group distribution, statistical significance, and predicted amino acid changes. *p*-values represent the significance of differences in SNP distributions between groups.

The discriminant analysis results for the association between gene types and healthy status were displayed in Table 3. The model correctly identified either healthy or affected cows in 100% of the situations, according to the organizational implications. The model’s SNP markers demonstrated a high level of discriminatory power, indicating their potential utility as genetic markers for milk fever susceptibility in cows.

**Table 3:** Discriminant analysis for classification of type of genes and healthy status of examined cows.

Actual group	Predicted healthy	Predicted milk fever	Total
Healthy	50	0	50
Milk fever	0	50	50

Overall classification accuracy = 100%.

### Findings of artificial neural networks

The examined genes were evaluated by artificial neural network analysis based on how well they predicted disease (Supplementary Figure S14). *NUAK1* had the highest normalized importance among all assessed biomarkers, followed by *NESP55*, suggesting better predictive performance for the classification of milk fever. With varying significance ratings, the remaining genes added to the predictive model. These results show that the identification of potential biomarkers linked to milk fever is significantly enhanced by combining targeted gene-expression biomarkers with machine-learning analysis.

## Discussion

As part of the investigation into molecular mechanisms involved in the pathogenesis of milk fever in Holstein dairy cows, the current study has used a combination of targeted blood gene-expression profiling, coding region SNP analysis and artificial neural networks (ANNs). In addition to coding region disease-specific SNPs, changes have been observed in the expression of genes involved in calcium signaling pathways, endocrine regulation, calcium intracellular transport and cellular metabolism. Further, *NUAK family kinase 1* (*NUAK1*) and *neuroendocrine secretory protein 55* (*NESP55*) were identified as the most effective disease-related biomarkers through ANN analysis. This emphasizes the need to incorporate genetic biomarkers into machine learning strategies for improving disease prediction.

The novelty of the current technique comes not only from using the individual techniques but through complementary usage of the methods for candidate marker selection. The targeted gene-expression profiling allowed identifying the molecular phenotypes of the clinical milk fever, whereas the coding region sequencing helped obtain data on the genetic variations within the candidate pathways. ANN analysis allowed a nonlinear ranking of the gene expression variables depending on their significance in classification and highlighted NUAK1 and NESP55 as the most informative candidates. In other words, this workflow combines the disease-related transcriptional signatures with the genetic variation and biomarker selection techniques. Nevertheless, due to the fact that the current study is conducted on a small population of one herd without any additional validation sample, the current results can be considered candidate markers.

The disease develops when the sudden increase in calcium requirements at the onset of breastfeeding exceeds the capacity of homeostatic regulatory mechanisms responsible for maintaining extracellular calcium levels (1, 2). Although the condition has typically been known to be a metabolic disorder, it is becoming clear that changes in calcium availability also have implications on immune cell functionality and endocrine regulation and intracellular signal transduction pathways (23). The findings in this study support this hypothesis through coordinated changes in the expression of calcium transport and calcium-regulated signaling genes.

Among the downregulated genes, intracellular calcium sensing and signal transduction are closely related to *calcium/calmodulin-dependent protein kinase II alpha (CAMK2A), calcium/calmodulin-dependent protein kinase IV (CAMK4), annexin A6 (ANXA6), calcium voltage-gated channel subunit alpha1 S (CACNA1S)*, and *vitamin D receptor (VDR)*. Many physiological functions, such as cell activation, metabolism, immune response, and control of gene activity, are managed by calcium/calmodulin-dependent protein kinases (24). Therefore, when calcium levels are low, reduced expression of these genes may show weakened calcium-dependent signaling in circulating blood cells. Likewise, the lower expression of CACNA1S, which codes for the voltage-dependent L-type calcium channel’s α1S subunit, matches earlier findings of reduced cell responsiveness and lower calcium influx in hypocalcemic dairy cows (25). Similar results have been linked to lower immune function and weakened leukocyte activity during the transition phase (23). The notable drop in VDR expression emphasizes the importance of vitamin D signaling in maintaining calcium balance. Many genes tied to intestinal calcium absorption, bone remodeling, and kidney calcium reabsorption are controlled by the active vitamin D receptor (26, 27). Lower VDR expression may increase the risk of clinical hypocalcemia by hindering the body’s response to falling calcium levels. Recent reports of similar decreases in VDR expression in sheep facing mineral shortages support the idea that this system is important for mineral metabolism across ruminants (28).

Conversely, cows with milk fever had considerably higher levels of *protein kinase inhibitor beta (PKIB), acid-sensing ion channel 2 (ASIC2), period circadian regulator 1 (PER1), NUAK family kinase 1 (NUAK1), syntaxin 1A (STX1A), sorcin (SRI),* and *inositol 1,4,5-trisphosphate receptor type 1 (ITPR1).* These genes are involved in energy metabolism, circadian rhythm, intracellular calcium release, and cellular tolerance to metabolic stress. During systemic hypocalcemia, increased expression of *ITPR1* and *SRI*, which both control the release of calcium from intracellular reserves, may be a compensatory reaction meant to preserve intracellular calcium signaling (29, 30). Similarly, as previously noted in cardiovascular and metabolic illnesses, elevated *STX1A* expression may represent adaptive responses to changes in calcium channel function and cellular stress (31, 32).

The significant overexpression of *NUAK1*, which also turned out to be the most significant biomarker in ANN analysis, was one of the most noteworthy discoveries. NUAK1 is a member of the AMPK-related kinase family and has a role in glucose metabolism, cellular energy sensing, and metabolic stress tolerance (33, 34). Increased *NUAK1* expression may indicate metabolic response to energy imbalance during early lactation, as cows with milk fever often have disruptions in glucose metabolism due to hypocalcemia (35). The fact that NUAK1 was shown to be the top biomarker further implies that the pathophysiology of milk fever is intimately related to energy metabolism.

In line with this, increased expression of *PER1* is physiologically reasonable because the release of glucocorticoids increases under hypocalcemia and physiological stress in parturition (36, 37). Thus, the activation of circadian genes may represent part of an endocrine response that follows metabolic adaptation at the time around parturition.

Sequence analysis identified twenty-three coding-region SNPs, including both synonymous and nonsynonymous changes, distributed throughout the genes examined. Nonsynonymous substitutions can change protein structure and have a direct impact on biological function, while synonymous variants leave the amino acid sequence unchanged but may affect mRNA stability, translational efficiency or transcript abundance (38, 39).

It may be necessary to further investigate whether genetic diversity in calcium-regulatory genes influences milk fever susceptibility because many SNP were significantly different between healthy and afflicted cows. The conservation of many of these variations among closely related ruminant species further supports the functional significance of these genomic regions (40).

The integration of molecular biomarkers with machine-learning analysis in this study was one of the main strengths. On the other hand, although classical statistical methods could detect significant differences among experimental groups, the complex nonlinear interactions among biological variables might not be adequately described. Conversely, ANN schemes simultaneously evaluate a number of competing and interacting biomarkers and rank them according to their predictive relevance. The identification of *NUAK1* and *NESP55* as the most informative biomarkers illustrates the application of machine-learning approaches to rank candidate biomarkers for further validation and enhanced milk fever classification. In conclusion, the amalgamation of computer prediction and transcriptome profiling is a promising strategy to beef-up precision medicine for cattle.

Functional characterization of the coding region variants allows for further biological understanding of the identified expression patterns (41). More specifically, the nonsynonymous variants that were found in *CACNA1S, PER1, NUAK1*, and *NESP55* led to amino acid changes and thus constitute reasonable candidates for the investigation of their functional significance. *CACNA1S* encodes the α1S subunit of a voltage-gated calcium channel and plays a critical role in calcium-dependent signaling (42). NUAK1 is an AMPK-related kinase involved in energy sensing in cells and metabolic adaptation; accordingly, the significant elevation in expression levels and normalized importance in ANN analysis could be anticipated (43). *PER1* is a gene that regulates circadian rhythm and might play a role in the process of endocrine and metabolic adaptation at the transitional period (44). NESP55 is a neuroendocrine secretory protein that is associated with regulated secretion and represents the second most informative biomarker in ANN analysis (45). While these mutations might lead to alterations in protein conformation or functionality, the functional impact of these variants could not be established on the basis of the sequence data only.

There are multiple limitations that need to be considered. Animals from a single herd were involved the candidate genes evaluated in this study were selected on the basis of their biological relevance. These biomarkers must be validated in larger, genetically diverse dairy populations prior to being employed in breeding or herd health applications. In addition, to demonstrate causal relationships between genetic variation and milk fever risk, functional studies investigating the biological consequences of identified SNPs are warranted. Furthermore, the perfect 100% classification in the current data set should not be considered as an indication for the same level of performance in independent herds, at different physiologic stages, or at other sampling times. Validation for temporal and external was not done. Future research is encouraged to assess the biomarkers in prospective studies using independent herds and lactation stages.

## Conclusion

In conclusion, our result demonstrated that the hypocalcemia risk of milk fever in Holstein dairy cows may be associated with pronounced coordination between positive and negative regulators of calcium signaling, vitamin D signaling, and energy metabolism at the transcriptional level and the coordinated expression of these genes involved in milk production. Using targeted gene-expression biomarkers, SNP analysis, and artificial neural network modeling, NUAK1 and NESP55 emerged as the best predictive biomarkers for the milk fever As such, the current results provide a framework for future studies on molecular biomarker identification, genomic prediction models and marker assisted breeding in other dairy cattle populations.

## Data Availability

The datasets presented in this study can be found in online repositories. The names of the repository/repositories and accession number(s) can be found in the article/Supplementary material.

## References

[1] Horst RL Goff JP Reinhardt TA. Adapting to the transition between gestation and lactation: differences between rat, human and dairy cow. J Mammary Gland Biol Neoplasia. (2005) 10:141–56. doi: 10.1007/s10911-005-5397-x, 16025221

[2] Goff JP. The monitoring, prevention, and treatment of milk fever and subclinical hypocalcemia in dairy cows. Vet J. (2008) 176:50–7. doi: 10.1016/j.tvjl.2007.12.020, 18342555

[3] Lee S Cho K Park M Choi T Kim SD Do CH. Genetic parameters of milk β-hydroxybutyric acid and acetone and their genetic association with milk production traits of Holstein cattle. Asian Australas J Anim Sci. (2016) 29:1530–40. doi: 10.5713/ajas.16.0310, 27608643 PMC5088371

[4] Seymour D Cánovas A Baes CF Chud STC Osborne VR Cant JP . Invited review: determination of large-scale individual dry matter intake phenotypes in dairy cattle. J Dairy Sci. (2019) 102:7655–63. doi: 10.3168/jds.2019-16454, 31255263

[5] Knop R Cernescu H. Effects of negative energy balance on reproduction in dairy cows. Lucrări. Stiinłifice. Med Vet. (2009) 42:198–205.

[6] Mulligan F O'Grady L Rice D Ml D. Production diseases of the transition cow: milk fever and subclinical hypocalcaemia. Iris Vet J. (2006) 59:697–701.

[7] Soares R Vargas G Muniz M Squires E. Differential gene expression in dairy cows under negative energy balance and ketosis: a systematic review and meta-analysis. J Dairy Sci. (2021) 104:602–15. doi: 10.3168/jds.2020-18883, 33189279

[8] Liang D Arnold L Stowe C Harmon RJ Bewley JM. Estimating US dairy clinical disease costs with a stochastic simulation model. J Dairy Sci. (2017) 100:1472–86. doi: 10.3168/jds.2016-11565 28012631

[9] Almeida PE Weber PS Burton JL Tempelman RJ Steibel JP Zanella AJ. Gene expression profiling of peripheral mononuclear cells in lame dairy cows with foot lesions. Vet Immunol Immunopath. (2007) 120:234–45. doi: 10.1016/j.vetimm.2007.06.028 17675248

[10] Liew CC Ma J Tang HC Zheng R Dempsey AA. The peripheral blood transcriptome dynamically reflects system wide biology: a potential diagnostic tool. J Lab Clin Med. (2006) 147:126–32. doi: 10.1016/j.lab.2005.10.005 16503242

[11] Lutzow YCS Donaldson L Gray CP Vuocolo T Pearson RD Reverter A . Identification of immune genes and proteins involved in the response of bovine mammary tissue to *Staphylococcus aureus* infection. BMC Vet Res. (2008) 4:18. doi: 10.1186/1746-6148-4-18, 18513449 PMC2430192

[12] Newman JH Holt TN Hedges LK. High-altitude pulmonary hypertension in cattle (brisket disease): candidate genes and gene expression profiling of peripheral blood mononuclear cells. Pulmo Circu. (2011) 1:462–9. doi: 10.4103/2045-8932.93545, 22530101 PMC3329076

[13] Ishida S Yonezawa T Eirai S Kizaki K Hashizume K Tomioka M . Hormonal differences in peripheral blood and gene profiling in the liver and lymphocytes in Japanese black cattle with growth retardation. J Vet Medi Sci. (2013) 75:17–25. doi: 10.1292/jvms.12-0141, 22878536

[14] Ginsburg GS Haga SB. Translating genomic biomarkers into clinically useful diagnostics. Exper Revi Mol Diagnos. (2006) 6:179–91. doi: 10.1586/14737159.6.2.179, 16512778

[15] Pryce J Parker Gaddis KL Koeck A Bastin C Abdelsayed M Gengler N . Invited review: opportunities for genetic improvement of metabolic diseases. J Dairy Sci. (2016) 99:6855–73. doi: 10.3168/jds.2016-10854 27372587

[16] Lean I DeGaris P McNeil D Block E. Hypocalcemia in dairy cows: meta-analysis and dietary cation anion difference theory revisited. J Dairy Sci. (2006) 89:669–84. doi: 10.3168/jds.S0022-0302(06)72130-0, 16428636

[17] Sun Y Xu C Li C Xia C Xu C Wu L . Characterization of the serum metabolic profile of dairy cows with milk fever using 1H-NMR spectroscopy. Vet Q. (2014) 34:159–63. doi: 10.1080/01652176.2014.924642, 24832124

[18] Meng C Luo P Ren W Xie X Zeng Y Wang J . Identification of candidate genes and metabolites associated with lactation performance in Kazakh mares using blood multi-omics and machine learning. BMC Genomics. (2026). doi: 10.1186/s12864-026-13131-w PMC13617780 42410339

[19] Pfaffl MW. A new mathematical model for relative quantification in real-time RT–PCR. Nucleic Acids Res. (2001) 29:e45. doi: 10.1093/nar/29.9.e45, 11328886 PMC55695

[20] Sanger F Nicklen S Coulson AR. DNA sequencing with chain-terminating inhibitors. Proc Natl Acad Sci USA. (1977) 74:5463–7. doi: 10.1073/pnas.74.12.5463, 271968 PMC431765

[21] Altschul SF Gish W Miller W Myers EW Lipman DL. Basic local alignment search tool. J Mol Biol. (1990) 215:403–10. doi: 10.1016/S0022-2836(05)80360-2, 2231712

[22] Tamura K Dudley J Nei M Kumar S. MEGA4: molecular evolutionary genetics analysis (MEGA) software version 4.0. Mol Biol Evol. (2007) 24:1596–9. doi: 10.1093/molbev/msm092, 17488738

[23] Kimura K Reinhardt T Goff J. Parturition and hypocalcemia blunts calcium signals in immune cells of dairy cattle. J Dairy Sci. (2006) 89:2588–95. doi: 10.3168/jds.S0022-0302(06)72335-9, 16772578

[24] Zhuang Y Liu P Zhang C Ye S Hu G Cao H. Effect of cadmium on the concentration of ceruloplasmin and its mRNA expression in goats under molybdenum stress. Pak Vet J. (2016) 36:209–13.

[25] Zhang B Ma X Huang B Jiang Q Loor JJ Lv X . Transcriptomics of circulating neutrophils in dairy cows with subclinical hypocalcemia. Front Vet Sci. (2022) 9:959831. doi: 10.3389/fvets.2022.959831 PMC9514324 36176696

[26] Haussler MR Whitfield GK Kaneko I Haussler CA Hsieh D Hsieh JC . Molecular mechanisms of vitamin D action. Calcif Tissue Int. (2013) 92:77–98. doi: 10.1007/s00223-012-9619-0, 22782502

[27] Nelson C Reinhardt T Thacker T Beitz DC Lippolis JD. Modulation of the bovine innate immune response by production of 1α, 25-dihydroxyvitamin D3 in bovine monocytes. J Dairy Sci. (2010) 93:1041–9. doi: 10.3168/jds.2009-2663, 20172224

[28] Emam R Ghanem PMM Abdel-Raof Y EL-khaiat HM Elsayed AA Helal MA. Gene expression of oxidative/antioxidative markers, VDR, CAMK4 and ceruloplasmin in Baladi sheep with minerals deficiency. Egypt J Vet Sci. (2025) 56:1121–33. doi: 10.21608/ejvs.2024.280240.1971

[29] Foskett JK White C Cheung KH Mak DOD. Inositol trisphosphate receptor Ca2+ release channels. Physiol Rev. (2007) 87:593–658. doi: 10.1152/physrev.00035.2006, 17429043 PMC2901638

[30] Oh-hora M Rao A. Calcium signaling in lymphocytes. Current Opinion Immunol. (2008) 20:250–8. doi: 10.1016/j.coi.2008.04.004, 18515054 PMC2574011

[31] Cao F Hata R Zhu P Niinobe M Sakanaka M. Up-regulation of syntaxin1 in ischemic cortex after permanent focal ischemia in rats. Brain Res. (2009) 1272:52–61. doi: 10.1016/j.brainres.2009.03.047, 19344701

[32] Chao C Liang T Kang Y Lin X Xie H Feng ZP . Syntaxin-1A inhibits K_ATP_ channels by interacting with specific conserved motifs within sulfonylurea receptor 2A. J Mol Cell Cardiol. (2011) 51:790–802. doi: 10.1016/j.yjmcc.2011.08.011, 21884702

[33] Humbert N Navaratnam N Augert A Costa MD Martien S Wang J . Regulation of ploidy and senescence by the AMPK-related kinase NUAK1. EMBO J. (2010) 29:376–86. doi: 10.1038/emboj.2009.342, 19927127 PMC2824453

[34] Inazuka F Sugiyama N Tomita M Abe T Shioi G Esumi H. Muscle-specific knock-out of NUAK family SNF1-like kinase 1 (NUAK1) prevents high fat diet-induced glucose intolerance. J Biol Chem. (2012) 287:16379–89. doi: 10.1074/jbc.M111.302687, 22418434 PMC3351321

[35] Larsen T Møller G Bellio R. Evaluation of clinical and clinical chemical parameters in periparturient cows. J Dairy Sci. (2001) 84:1749–58. doi: 10.3168/jds.S0022-0302(01)74610-3, 11467825

[36] Gery S Komatsu N Baldjyan L Yu A Koo D Koeffler HP. The circadian gene per1 plays an important role in cell growth and DNA damage control in human cancer cells. Mol Cell. (2006) 22:375–82. doi: 10.1016/j.molcel.2006.03.038, 16678109

[37] Nebzydoski SJ Pozzo S Nemec L Rankin MK Gressley TF. The effect of dexamethasone on clock gene mRNA levels in bovine neutrophils and lymphocytes. Vet Immunol Immunopathol. (2010) 138:183–92. doi: 10.1371/journal.pone.0055894 20807668

[38] Barton NH. Mutation and the evolution of recombination. Philos Trans R Soc B Biol Sci. (2010) 365:1281–94. doi: 10.1098/rstb.2009.0320, 20308104 PMC2871826

[39] Dakal TC Kala D Dhiman G Yadav V Krokhotin A Dokholyan NV. Predicting the functional consequences of non-synonymous single nucleotide polymorphisms in IL8 gene. Sci Rep. (2017) 7:6525. doi: 10.1038/s41598-017-06575-4, 28747718 PMC5529537

[40] Retief A Brito LF Visser C. Development history, genomic diversity, population structure, and selection signatures in worldwide Boer goat populations. Animal. (2026) 20:101901. doi: 10.1016/j.animal.2026.101901 42531739

[41] Pilalis E Zisis D Andrinopoulou C Karamanidou T Antonara M Stavropoulos TG . Genome-wide functional annotation of variants: a systematic review of state-of-the-art tools, techniques and resources. Front Pharmacol. (2025) 16:1474026. doi: 10.3389/fphar.2025.1474026, 40098614 PMC11911558

[42] Sangkuhl K Dirksen RT Alvarellos ML Altman RB Klein TE. PharmGKB summary: very important pharmacogene information for CACNA1S. Pharmacogenet Genomics. (2020) 30:34–44. doi: 10.1097/FPC.0000000000000393, 31851124 PMC7008936

[43] Escalona E Muñoz M Pincheira R Elorza ÁA Castro AF. Cytosolic NUAK1 enhances ATP production by maintaining proper glycolysis and mitochondrial function in cancer cells. Front Oncol. (2020) 10:1123. doi: 10.3389/fonc.2020.01123, 32754444 PMC7367139

[44] Zhao Y Li Y Lu T Huang X Chen K Tan L . Research progress on the role and mechanism of circadian clock gene PER1 in the occurrence and development of multiple diseases. Front Immunol. (2025) 16:1715457. doi: 10.3389/fimmu.2025.1715457, 41451215 PMC12727441

[45] Srivastava A Padilla O Fischer-Colbrie R Tischler AS Dayal Y. Neuroendocrine secretory protein-55 (NESP-55) expression discriminates pancreatic endocrine tumors and pheochromocytomas from gastrointestinal and pulmonary carcinoids. Am J Surg Pathol. (2004) 28:1371–8. doi: 10.1097/01.pas.0000135527.96318.20, 15371954

