# Supplementary material for: Integrative targeted blood gene-expression profiling, coding-region genetic variants, and machine learning identify candidate biomarkers of milk fever in Holstein dairy cattle
Source: Front Vet Sci. 2026 Sep 21;13:1946092. doi: 10.3389/fvets.2026.1946092 (PMC13634804; doi:10.3389/fvets.2026.1946092)
Supplement: Supplementary file 1 [file Data_Sheet_1.PDF]

```
NM_001075938.1      CCAGAAGTGCTGCGGAAGGACCCGTATGGGAAGCCCGTGGACCTGTGGGCCTGCGGGGTC   60
H                   CCAGAAGTGCTGCGGAAGGACCCGTATGGGAAGCCCGTGGACCTGTGGGCCTGCGGGGTC   60
MF                  CCAGAAGTGCTGCGGAAGGACCCGTATGGGAAGCCCGTGGACCTGTGGGCCTGCGGGGTC   60
                    ************************************************************

NM_001075938.1      ATCCTGTACATCCTGCTGGTGGGGTACCCCCCGTTCTGGGATGAGGACCAGCATCGCCTG   120
H                   ATCCTGTACATCCTGCTGGTGGGGTACCCCCCGTTCTGGGATGAGGACCAGCATCGCCTG   120
MF                  ATCCTGTACATCCTGCTGGTGGGGTACCCCCCGTTCTGGGATGAGGACCAGCATCGCCTG   120
                    ************************************************************

NM_001075938.1      TACCAGCAGATCAAAGCTGGCGCCTATGATTTCCCGTCACCAGAATGGGACACGGTTACC   180
H                   TACCAGCAGATCAAAGCTGGCGCCTATGATTTCCCGTCACCAGAATGGGACACCGTTACC   180
MF                  TACCAGCAGATCAAAGCTGGCGCCTATGATTTCCCGTCACCAGAATGGGACACGGTTACC   180
                    ***************************************************** ******

NM_001075938.1      CCGGAAGCCAAGGATCTGATCAATAAGATGCTGACCATCAACCCATCCAAACGCATCACG   240
H                   CCAGAAGCCAAGGATCTGATCAATAAGATGCTGACCATCAACCCATCCAAACGCATCACG   240
MF                  CCGGAAGCCAAGGATCTGATCAATAAGATGCTGACCATCAACCCATCCAAACGCATCACG   240
                    ** *********************************************************

NM_001075938.1      GCCGCTGAGGCCCTCAAGCACCCCTGGATCTCGCACCGGTCCACCGTGGCCTCCTGCATG   300
H                   GCCGCTGAGGCCCTCAAGCACCCCTGGATCTCGCACCGGTCCACCGTGGCCTCCTGCATG   300
MF                  GCCGCTGAGGCCCTCAAGCACCCCTGGATCTCGCACCGGTCCACCGTGGCCTCCTGCATG   300
                    ************************************************************

NM_001075938.1      CACAGACAGGAGACCGTGGACTGCCTGAAGAAGTTCAACGCCAGGAGAAAACTGAAGGGA   360
H                   CACAGACAGGAGACCGTGGACTGCCTGAAGAAGTTCAACGCCAGGAGAAAACTGAAGGGA   360
MF                  CACAGACAGGAGACCGTGGACTGCCTGAAGAAGTTCAACGCCAGGAGAAAACTGAAGGGA   360
                    ************************************************************

NM_001075938.1      GCCATCCTCACCACTATGCTGGCCACCAGGAACTTCTCTGGCGGGAAGAGTGGAGGAAAC   420
H                   GCCATCCTCACCACTATGCTGGCCACCAGGAACTTCTCTGGCGGGAAGAGTGGAGGAAAC   420
MF                  GCCATCCTCACCACTATGCTGGCCACCAGGAACTTCTCTGGCGGGAAGAGTGGAGGAAAC   420
                    ************************************************************

NM_001075938.1      AAGAAGAACGATGGTGTGAAGGAATCTTCAGAGAGCACCAATACCACCATTGAGGA       476
H                   AAGAAGAACGATGGTGTGAAGGAATCTTCAGAGAGCACCAATACCACCATTGAGGA       476
MF                  AAGAAGAACGATGGTGTGAAGGAATCTTCAGAGAGCACCAATACCACCATTGAGGA       476
                    ********************************************************
```

Figure S1: The alignment of the CAMK2A gene (476 bp) in healthy dairy cows (H) and those with milk fever (MF).

```
NM_001103224.1      ACCGATGAGAAGTGCCTCATTGAGATCTTGGCTTCTCGGACCAACGAGCAGATCCACCAG   60
H                   ACCGATGAGAAGTGCCTCATTGAGATCTTGGCTTCTCGGACCAACGAGCAGATCCACCAG   60
MF                  ACCGATGAGAAGTGCCTCATTGAGATCTTGGCTTCTCGGACCAACGAGCAGATCCACCAG   60
                    ************************************************************

NM_001103224.1      CTGGTGGCAGCATACAAAGATGCCTACGAGCGAGACCTGGAGGCTGACATCACTGGGGAC   120
H                   CTGGTGGCAGCATACAAAGATGCCTACGAGCGAGACCTGGAGGCTGACATCACTGGGGAC   120
MF                  CTGGTGGCAGCATACAAAGATGCCTACGAGCGAGACCTGGAGGCTGACATCACTGGGGAC   120
                    ************************************************************

NM_001103224.1      ACCTCTGGCCACTTCCGGAAGATGCTCGTGGTCCTGCTGCAGGGTACCAGGGAGGAGGAT   180
H                   ACCTCTGGCCACTTCCGGAAGATGCTCGTGGTCCTGCTGCAGGGTACCAGGGAGGAGGAT   180
MF                  ACCTCTGGCCACTTCCGGAAGATGCTCGTGGTCCTGCTGCAGGGTACCAGGGAGGAGGAT   180
                    ************************************************************

NM_001103224.1      GACGTAGTGAGCGAGGACTTGGTGCAGCAGGACGTCCAGGACCTGTATGAGGCAGGGGAA   240
H                   GACGTAGTGAGCGAGGACTTGGTGCAGCAGGACGTCCAGGACCTGTATGAGGCAGGGGAA   240
MF                  GACGTAGTGAGCGAGGACTTGGTGCAGCAGGACGTTCAGGACCTGTATGAGGCAGGGGAA   240
                    *********************************** ************************

NM_001103224.1      CTGAAATGGGGAACAGATGAAGCCCAGTTCATTTACATTTTGGGAAATCGCAGCAAGCAG   300
H                   CTGAAATGGGGAACAGATGAAGCCCAGTTCATTTACATTTTGGGAAATCGCAGCAAGCAG   300
MF                  CTGAAATGGGGAACAGATGAAGCCCAGTTCATTTACATTTTGGGAAATCGCAGCAAGCAG   300
                    ************************************************************

NM_001103224.1      CATCTCCGGTTGGTATTTGATGAGTATCTGAAGACCACGGGGAAGCCGATTGAAGCCAGC   360
H                   CATCTCCGGTTGGTATTTGATGAGTATCTGAAGACCACGGGGAAGCCGATTGAAGCCAGC   360
MF                  CATCTCCGGTTGGTATTTGATGAGTATCTGAAGACCACGGGGAAGCCGATTGAAGCCAGC   360
                    ************************************************************

NM_001103224.1      ATCCGA  366
H                   ATCCGA  366
MF                  ATCCGA  366
                    ******
```

Figure S2: The alignment of the ANXA6 gene (366 bp) aligns in healthy dairy cows (H) and those with milk fever (MF).

```
XM_059875843.1      ACTGAAATTGAGATGGAGGAGATGGAATCGCCCGTCTTCCTGGAGGACTTCCCTCAACGT   60
H                   ACTGAAATTGAGATGGAGGAGATGGAATCGCCCGTCTTCCTGGAGGATTTCCCTCAACGT   60
MF                  ACTGAAATTGAGATGGAGGAGATGGAATCGCCCGTCTTCCTGGAGGACTTCCCTCAACGT   60
                    *********************************************** ************

XM_059875843.1      CCGAGTGCCCACCCCCTTGCTCGTGCCAACACCAACAACGCCAACGCCAACGTCGCCAGT   120
H                   CCGAGTGCCCACCCCCTTGCTCGTGCCAACACCAACAACGCCAACGCCAACGTCGCCAGT   120
MF                  CCGAGTGCCCACCCCCTTGCTCGTGCCAACACCAACAATGCCAACGCCAACGTCGCCAGT   120
                    ************************************** *********************

XM_059875843.1      GGCAACGGCAACCATGGCAACAGCCCAGCATTTCCCAGCCCCATAGATAGCAGACCACCA   180
H                   GGCAACGGCAACCATGGCAACAGCCCAGCATTTCCCAGCCCCATAGATAGCAGACCACCA   180
MF                  GGCAACGGCAACCATGGCAACAGCCCAGCATTTCCCAGCCCCATAGATAGCAGACCACCA   180
                    ************************************************************

XM_059875843.1      GGCTCCCCATCCCTGGGATTCTCCAGGCAAGAACACTGGAGTGGGTTGCCATTTTCTTCT   240
H                   GGCTCCCCATCCCTGGGATTCTCCAGGCAAGAACACTGGAGTGGGTTGCCATTTTCTTCT   240
MF                  GGCTCCGCATCCCTGGGATTCTCCAGGCAAGAACACTGGAGTGGGTTGCCATTTTCTTCT   240
                    ****** *****************************************************

XM_059875843.1      TCAGTACGTGAAATTGAAGTCGCCCAGTCATGTCTGACTCTAAGCAACCCATGGACTGCA   300
H                   TCAGTACGTGAAATTGAAGTCGCCCAGTCATGTCTGACTCTAAGCAACCCATGGACTGCA   300
MF                  TCAGTACGTGAAATTGAAGTCGCCCAGTCATGTCTGACTCTAAGCAACCCATGGACTGCA   300
                    ************************************************************

XM_059875843.1      GCCCACCAGGCTCCTCCATCCATGGGATTTTCCAGGCAAGAGTACTGGAGTGGGGTGCCA   360
H                   GCCCACCAGGCTCCTCCATCCATGGGATTTTCCAGGCAAGAGTACTGGAGTGGGGTGCCA   360
MF                  GCCCACCAGGCTCCTCCATCCATGGGATTTTCCAGGCAAGAGTACTGGAGTGGGGTGCCA   360
                    ************************************************************

XM_059875843.1      TTGCCTTCTCCGAGCTGCGGACTGGGTATAATCTGTGATCGAGTGCCAGGGATCGAGGGA   420
H                   TTGCCTTCTCCGAGCTGCGGACTGGGTATAATCTGTGATCGAGTGCCAGGGATCGAGGGA   420
MF                  TTGCCTTCTCCGAGCTGCGGACTGGATATAATCTGTGATCGAGTGCCAGGGATCGAGGGA   420
                    ************************* **********************************

XM_059875843.1      TGAACAGAGAGGGCCTTG        438
H                   TGAACAGAGAGGGCCTTG        438
MF                  TGAACAGAGAGGGCCTTG        438
                    ******************
```

Figure S3: The alignment of the CACNA1S gene (438 bp) aligns in healthy dairy cows (H) and those with milk fever (MF).

```
NM_001167932.2      CACAAGACCTACGACGACACCTACTCCGACTTCAGCCAGTTCCGGCCTCCAGTTCGCAAC   60
H                   CACAAGACCTACGACGACACCTACTCCGACTTCAGCCAGTTCCGGCCTCCAGTTCGCAAC   60
MF                  CACAAGACCTACGACGACACCTACTCCGACTTCAGCCAGTTCCGGCCTCCAGTTCGCAAC   60
                    ************************************************************

NM_001167932.2      AGTGAGGACGAGGGGAACCGTCCTTTGAGGTCGATACTCACGCCCAGCTTCTCTGGAAAC   120
H                   AGTGAGGACGAGGGGAACCGTCCTTTGAGGTCGATACTCACGCCCAGCTTCTCTGGAAAC   120
MF                  AGTGAGGACGAGGGGAACCGTCCTTTGAGGTCGATACTCACGCCCAGCTTCTCTGGAAAC   120
                    ************************************************************

NM_001167932.2      TCATCCTCCTCCTGTTCGGATCACTGTACCTCCTCTCCAGACACAATGGAGCCAACCAGC   180
H                   TCATCCTCCTCCTGTTCGGATCACTGTACCTCCTCTCCAGACACAATGGAGCCAACCAGC   180
MF                  TCATCCTCCTCCTGTTCGGATCACTGTACCTCCTCTCCAGACACAATGGAGCCAACCAGC   180
                    ************************************************************

NM_001167932.2      TTCTCCAACCAGGATCTGAATGAAGAAGACTCCGATGACCCTTCTGTGACCCTGGACCTG   240
H                   TTCTCCAACCAGAATCTGAATGAAGAAGACTCCGATGACCCTTCTGTGACCCTGGACCTG   240
MF                  TTCTCCAACCAGGATCTGAATGAAGAAGACTCCGATGACCCTTCTGTGACCCTGGACCTG   240
                    ************ ***********************************************

NM_001167932.2      TCCCAGCTCTCCATGCTGCCCCACCTGGCTGACCTGGTCAGTTATAGCATCCAGAAGGTC   300
H                   TCCCAGCTCTCCATGCTGCCCCACCTGGCTGACCTGGTCAGTTACAGCATCCAGAAGGTC   300
MF                  TCCCAGCTCTCCATGCTGCCCCACCTGGCTGACCTGGTCAGTTATAGCATCCAGAAGGTC   300
                    ******************************************** ***************

NM_001167932.2      ATCGGCTTTGCCAAGATGATCCCAGGGTTCAGAGACCTCACCCCTGAGGACCAGATCGTG   360
H                   ATCGGCTTTGCCAAGATGATCCCAGGGTTCAGAGACCTCACCCCTGAGGACCAGATCGTG   360
MF                  ATCGGCTTTGCCAAGATGATCCCAGGGTTCAGAGACCTCACCCCTGAGGACCAGATCGTG   360
                    ************************************************************

NM_001167932.2      CTGCTGAAGTCGAGTGCCATTGAAGTCATCATGCTTCGCTCCAACCAGTCCTTCACCCTG   420
H                   CTGCTGAAGTCGAGTGCCATTGAAGTCATCATGCTTCGCTCCAACCAGTCCTTCACCCTG   420
MF                  CTGCTGAAGTCGAGTGCCATTGAAGTCATCATGCTGCGCTCCAACCAGTCCTTCACCCTG   420
                    *********************************** ************************

NM_001167932.2      GACGACATGTCCTGGACCTGTGGCAGCCCGGACTACAAGTACCAAGTCAGCG  472
H                   GACGACATGTCCTGGACCTGTGGCAGCCCGGACTACAAGTACCAAGTCAGCG  472
MF                  GACGACATGTCCTGGACCTGTGGCAGCCCGGACTACAAGTACCAAGTCAGCG  472
                    ****************************************************
```

Figure S4: The alignment of the VDR gene (472 bp) aligns in healthy dairy cows (H) and those with milk fever (MF).

```
ON961763.1      GTAGAACATCAAGTACTCATGAAGACAGTATGTGGAACCCCAGGGTACTGCGCACCTGAA      60
H               GTAGAACATCAAGTACTCATGAAGACAGTATGTGGAACCCCAGGGTACTGCGCACCTGAA      60
MF              GTAGAACATCAAGTACTCATGAAGACAGTATGTGGAACCCCAGGGTACTGCGCACCTGAA      60
                ************************************************************

ON961763.1      ATCCTTAGAGGCTGTGCCTACGGACCCGAGGTGGACATGTGGTCTGTGGGGATAATCACC      120
H               ATCCTTAGAGGCTGTGCCTACGGACCCGAGGTGGACATGTGGTCTGTGGGAATAATCACC      120
MF              ATCCTTAGAGGCTGTGCCTACGGACCCGAGGTGGACATGTGGTCTGTGGGGATAATCACC      120
                ************************************************** *********

ON961763.1      TACATTTTACTTTGTGGATTTGAACCATTCTATGATGAGAGAGGTGATCAATTCATGTTC      180
H               TACATTTTACTTTGTGGATTTGAACCATTCTATGATGAGAGAGGTGATCAATTCATGTTC      180
MF              TACATTTTACTTTGTGGATTTGAACCATTCTATGATGAGAGAGGTGATCAATTCATGTTC      180
                ************************************************************

ON961763.1      AGGAGAATTCTGAATTGTGAATATTACTTTATCTCCCCCTGGTGGGATGAAGTATCTCTG      240
H               AGGAGAATTCTGAATTGTGAATATTACTTTATCTCCCCCTGGTGGGATGAAGTATCTCTG      240
MF              AGGAGAATTCTGAATTGTGAATATTACTTTATCTCCCCCTGGTGGGATGAAGTATCTCTG      240
                ************************************************************

ON961763.1      AATGCCAAGGATCTGGTCAGAAAATTAATTGTGTTGGATCCTAAGAAAAGGCTGACTACC      300
H               AATGCCAAGGATCTGGTCAGAAAATTAATTGTGTTGGATCCTAAGAAAAGGCTGACTACC      300
MF              AATGCCAAGGATCTGGTCAGAAAATTAATTGTGTTGGATCCTAAGAAAAGGCTGACTACC      300
                ************************************************************

ON961763.1      TTTCAAGCTCTCCAGCACCCGTGGGTCACAGGTAAAGCTGCCAATTTTGTACACATGGAT      360
H               TTTCAAGCTCTCCAGCACCCGTGGGTCACAGGTAAAGCTGCCAATTTTGTACACATGGAT      360
MF              TTTCAAGCTCTCCAGCACCCGTGGGTCACAGGTAAAGCTGCCAATTTTGTACACATGGAT      360
                ************************************************************

ON961763.1      ACGGCTCAAAAGAAGCTCCAAGAATTCAATGCCCGGCGCAAGCTCAAGGCGGCGGTCAAG      420
H               ACGGCTCAAAAGAAGCTCCAAGAATTCAATGCCCGGCGCAAGCTCAAGGCGGCGGTCAAG      420
MF              ACGGCTCAAAAGAAGCTCCAAGAATTCAATGCCCGGCGCAAGCTCAAGGCGGCGGTCAAG      420
                ************************************************************

ON961763.1      GCCGTGGTGGCCTCCTCCAGGCTCGGCAGCGCCAGCAGCAGCCACAGCGGCGTCCAGGA       479
H               GCCGTGGTGGCCTCCTCCAGGCTCGGCAGCGCCAGCAGCAGCCACAGCGGCGTCCAGGA       479
MF              GCCGTGGTGGCCTCCTCCAGGCTCGGCAGCGCCAGCAGCAGCCACAGCGGCGTCCAGGA       479
                ***********************************************************
```

Figure S5: The alignment of the CAMK4 gene (479 bp) aligns in healthy dairy cows (H) and those with milk fever (MF).

```
NM_001114518.2      ATGAGGACAGATTCACCCAAAATGACGGATGTGGAGCCTGTGGTCAACAATTTTGCGTCA   60
H                   ATGAGGACAGATTCACCCAAAATGACGGATGTGGAGCCTGTGGTCAACAATTTTGCGTCA   60
MF                  ATGAGGACAGATTCACCCAAAATGACGGATGTGGAGCCTGTGGTCAACAATTTTGCGTCA   60
                    ************************************************************

NM_001114518.2      TCAGCACGGGCAGGTCGCCGAAATGCTGTCCCAGACATCCAGGGTTCAACGGCTGCAGGC   120
H                   TCAGCACGGGCAGGTCGCCGAAATGCTGTCCCAGACATCCAGGGTTCAACGGCTGCAGGC   120
MF                  TCAGCACGGGCAGGTCGCCGAAATGCTGTCCCAGACATCCAAGGTTCAACGGCTGCAGGC   120
                    ***************************************** ******************

NM_001114518.2      GGGACCTTGGAGGAGTTGCCCGTCAAACTGGAAGCCCTATCCGTGAAGGAAGATGTGAAA   180
H                   GGGACCTTGGAGGAGTTGCCCGTCAAACTGGAAGCCCTATCCGTGAAGGAAGATGTGAAA   180
MF                  GGGACCTTGGAGGAGTTGCCCGTCAAACTGGAAGCCCTATCCGTGAAGGAAGATGTGAAA   180
                    ************************************************************

NM_001114518.2      AAGAAAGATGAAGAAACAGCACAAGATCAATTGGAAAAGCCCAAAGATGAAGGA234
H                   AAGAAAGATGAAGAAACAGCACAAGATCAATTGGAAAAGCCCAAAGATGAAGGA234
MF                  AAGAAAGATGAAGAAACAGCACAAGATCAATTGGAAAAGCCCAAAGATGAAGGA234
                    ******************************************************
```

Figure S6: The alignment of the PKIB gene (234 bp) aligns in healthy dairy cows (H) and those with milk fever (MF).

```
NM_001076484.1      ATCATGCTGGACATTCAGCAGGATGAGTACCTGCCCATCTGGGGCGAGACAGAGGAAACG   60
H                   ATCATGCTGGACATTCAGCAGGATGAGTACCTGCCCATCTGGGGCGAGACAGAGGAAACG   60
MF                  ATCATGCTGGACATTCAGCAGGATGAGTACCTGCCCATCTGGGGCGAGACAGAGGAAACG   60
                    ************************************************************

NM_001076484.1      ACATTTGAAGCAGGCGTGAAGGTTCAGATCCACAGTCAGTCTGAGCCGCCTTTCATCCAA   120
H                   ACATTTGAAGCAGGCGTGAAGGTTCAGATCCACAGTCAGTCTGAGCCGCCTTTCATCCAA   120
MF                  ACATTTGAAGCAGGCGTGAAGGTTCAGATCCACAGTCAGTCTGAGCCGCCTTTCATCCAA   120
                    ************************************************************

NM_001076484.1      GAGCTGGGCTTTGGTGTGGCACCAGGGTTCCAGACTTTTGTGGCCACACAGGAGCAGAGG   180
H                   GAGCTGGGCTTTGGTGTGGCACCAGGGTTCCAGACTTTTGTGGCCACACAGGAGCAGAGG   180
MF                  GAGCTGGGCTTTGGTGTGGCACCAGGGTTCCAGACTTTCGTGGCCACACAGGAGCAGAGG   180
                    ************************************** *********************

NM_001076484.1      CTCACATACCTGCCCCCGCCGTGGGGCGAGTGCCGGTCCTCCGAGATGGGGCTCGACTTC   240
H                   CTCACATACCTGCCCCCGCCGTGGGGCGAGTGCCGGTCCTCCGAGATGGGGCTCGACTTC   240
MF                  CTCACATACCTGCCCCCGCCGTGGGGCGAGTGCCGGTCCTCCGAGATGGGGCTCGACTTC   240
                    ************************************************************

NM_001076484.1      TTCCCTGTTTACAGCATCACCGCCTGTAGGATCGACTGTGAGACCCGCTACATTGTGGAG   300
H                   TTCCCTGTTTACAGCATCACCGCCTGTAGGATCGACTGTGAGACCCGCTACATCGTGGAG   300
MF                  TTCCCTGTTTACAGCATCACCGCCTGTAGGATCGACTGTGAGACCCGCTACATTGTGGAG   300
                    ***************************************************** ******

NM_001076484.1      AACTGCAACTGTCGCATGGTCCACATGCCAGGGGATGCCCCTTTCTGTACCCCTGAGCAG   360
H                   AACTGCAACTGTCGCATGGTCCACATGCCAGGGGATGCCCCTTTCTGTACCCCTGAGCAG   360
MF                  AACTGCAACTGTCGCATGGTCCACATGCCAGGGGATGCCCCTTTCTGTACCCCTGAGCAG   360
                    ************************************************************

NM_001076484.1      CACAAGGAGTGTGCAGAGCCTG    382
H                   CACAAGGAGTGTGCAGAGCCTG    382
MF                  CACAAGGAGTGTGCAGAGCCTG    382
                    **********************
```

Figure S7: The alignment of the ASIC2 gene (382 bp) aligns in healthy dairy cows (H) and those with milk fever (MF).

```
XM_059878047.1      GAGCAGGCCTTCCTCAGCCGCTTCCGTGACTTCAGCAGGCTGCGTGGACTGGACAGCTCC   60
H                   GAGCAGGCCTTCCTCAGCCGCTTCCGTGACTTCAGCAGGCTGCGTGGACTGGACAGCTCC   60
MF                  GAGCAGGCCTTCCTCAGCCGCTTCCGTGACTTCAGCAGGCTGCGTGGACTGGACAGCTCC   60
                    ************************************************************

XM_059878047.1      TCCACAGCCCCCTCGGCCCCTGGCGAGCGAGGCTGCCACCACAGCCTTGCCATCCCCGGC   120
H                   TCCACAGCCCCCTCGGCCCCTGGCGAGCGAGGATGCCACCACAGCCTTGCCATCCCCGGC   120
MF                  TCCACAGCCCCCTCGGCCCCTGGCGAGCGAGGCTGCCACCACAGCCTTGCCATCCCCGGC   120
                    ******************************** ***************************

XM_059878047.1      CGCCGCCACCACTGCCGATCCAAAGCCAAGCGCTCACGTCACCACCAGACAACTCGGGCC   180
H                   CGCCGCCACCACTGCCGATCCAAAGCCAAGCGCTCACGTCACCACCAGACAACTCGGGCC   180
MF                  CGCCGCCACCACTGCCGATCCAAAGCCAAGCGCTCACGTCACCACCAGACAACTCGGGCC   180
                    ************************************************************

XM_059878047.1      GAAGCCCCCGGCTACATCTCCCAGCCCTCGCCTGTGCCACCCTCTGCCCCCTGGCCCCCG   240
H                   GAAGCCCCCGGCTACATCTCCCAGCCCTCGCCTGTGCCACCCTCTGCCCCCTGGCCCCCG   240
MF                  GAAGCCCCCGGCTACATCTCCCAGCCCTCGCCTGTGCCACCCTCTGCCCCCTGGCCCCCG   240
                    ************************************************************

XM_059878047.1      CCACCAGCCACTCCTCCCTTCCCAGCTGTGGTCCAGCCATACCCACTCCCCGTGTTCCCC   300
H                   CCACCAGCCACTCCTCCCTTCCCAACTGTGGTCCAGCCATACCCACTCCCCGTGTTCCCC   300
MF                  CCACCAGCCACTCCTCCCTTCCCAGCTGTGGTCCAGCCATACCCACTCCCCGTGTTCCCC   300
                    *********************** ************************************

XM_059878047.1      CCGAGAGGTGGCTCTCAGCCTCTCCCTCCTGCTCCCACGTCTGTGCCTCCTGCAGCTTTC   360
H                   CCGAGAGGTGGCCCTCAGCCTCTCCCTCCTGCTCCCACGTCTGTGCCTCCTGCAGCTTTC   360
MF                  CCGAGAGGTGGCTCTCAGCCTCTCCCTCCTGCTCCCACGTCTGTGCCTCCTGCAGCTTTC   360
                    ************ ***********************************************

XM_059878047.1      CCTGCCCCCCTGGTGACCCCCATGGTGGCCTTGGTGCTCCCTAACTATCTGTTC414
H                   CCTGCCCCCCTGGTGACCCCCATGGTGGCCTTGGTGCTCCCTAACTATCTGTTC414
MF                  CCTGCCCCCCTGGTGACCCCCATGGTGGCCTTGGTGCTCCCTAACTATCTGTTC414
                    ******************************************************
```

Figure S8: The alignment of the PER1 gene (414 bp) aligns in healthy dairy cows (H) and those with milk fever (MF).

```
NM_001205496.1      GTGCTGGAGCGTCAGCGGTCGCTGAAGAAGTCCAAGAAAGAGAACGACTTCCCTCAGTCT    60
H                   GTGCTGGAGCGTCAGCGGTCGCTGAAGAAGTCCAAGAAAGAGAACGACTTCCCTCAGTCT    60
MF                  GTGCTGGAGCGTCAGCGGTCGCTGAAGAAGTCCAAGAAAGAGAACGACTTCCCTCAGTCT    60
                    ************************************************************

NM_001205496.1      GGCCAGGACTCGGTGCCCGAGAGCCCATCCAAGCTGAGCTCCAAGCGGCCCAAAGGGATC    120
H                   GGCCAGGACTCGGTGCCCGAGAGCCCATCCAAGCTGAGCTCCAAGCGGCCCAAAGGGATC    120
MF                  GGCCAGGACTCGGTGCCCGAGAGCCCATCCAAGCTGAGCTCCAAGCGGCCCAAAGGGATC    120
                    ************************************************************

NM_001205496.1      CTGAAGAAGCGGAGCAACAGCGAGCATCGCTCCCACAGCACTGGCTTCATTGAGGGTGTC    180
H                   CTGAAGAAGCGGAGCAACAGCGAGCATCGCTCCCACAGCACTGGCTTCATTGAGGGTGTC    180
MF                  CTGAAGAAGCGGAGCAACAGCGAGCATCGCTCCCACAGCACTGGCTTCATTGAGGGTGTC    180
                    ************************************************************

NM_001205496.1      GTCAGCCCGGCCTTACCACCCACTTTCAAGCTGGAACAGGACTTGTGCCGGACTGCTGTG    240
H                   GTCAGCCCGGCCTTACCACCCACTTTCAAGCTGGAACAGGACTTGTGCCGGACTGCTGTG    240
MF                  GTCAGCCCGGCCTTACCACCCACTTTCAAGCTGGAACAGGACTTGTGCCGGACTGCTGTG    240
                    ************************************************************

NM_001205496.1      CCCCTGCCAAGCCCCCCAGAGGCCGAAGTGCCAGGTAAACTCAGCCCCAAACAGTCGGCC    300
H                   CCCCTGCCAAGCCCCCCAGAGGCCGAAGTGCCAGGTAAACTCAGCCCCAAACAGTCGGCC    300
MF                  CCCCTGCCAAGCCCCCCAGAGGCCGAAGCGCCAGGTAAACTCAGCCCCAAACAGTCGGCC    300
                    **************************** *******************************

NM_001205496.1      ACGATGCCCAAGAAAGGCATCCTGAAAAAGACCCAGCAGAGAGAATCGGGTTACTATTCA    360
H                   ACGATGCCCAAGAAAGGCATCCTGAAAAAGACCCAGCAGAGAGAATCGGGTTACTATTCA    360
MF                  ACGATGCCCAAGAAAGGCATCCTGAAAAAGACCCAGCAGAGAGAATCGGGTTACTATTCA    360
                    ************************************************************

NM_001205496.1      TC      362
H                   TC      362
MF                  TC      362
                    **
```

Figure S9: The alignment of the NUAK1 gene (362 bp) aligns in healthy dairy cows (H) and those with milk fever (MF).

```
U77614.1      GATCGTAGATCCCGACCTCAGCTAGGGCGCCGAGCTCGCCACAATTACAACGATCTGTGC60
H             GATCGTAGATCCCGACCTCAGCTAGGGCGCCGAGCTCGCCACAATTACAACGATCTGTGC60
MF            GATCGTAGATCCCGACCTCAGCTAGGGCGCCGAGCTCGCCACAATTACAACGATCTGTGC60
              ************************************************************

U77614.1      CCACCCATAGGCCGCCGGGCAGCCACCGCGCTCCTCTGGCTCTCCTGCTCCATCGCGCTC120
H             CCACCCATAGGCCGCCGGGCAGCCACCGCGCTCCTCTGGCTCTCCTGCTCCATCGCGCTC120
MF            CCACCCATAGGCCGCCGGGCAGCCACCGCGCTCCTCTGGCTCTCCTGCTCCATCGCGCTC120
              ************************************************************

U77614.1      CTCCGCGCCCTTGCCACCTCCAGCACCCGCGCCCAGCAGCGCGCGGCTGCCCAGCGCCGG180
H             CTCCGCGCCCTTGCCACCTCCAGCACCCGCGCCCAGCAGCGCGCGGCTGCCCAGCGCCGG180
MF            CTCCGCGCCCTTGCCACCTCCAGCACCCGCGCCCAGCAGCGCGCGGCTGCCCAGCGCCGG180
              ************************************************************

U77614.1      ACCTTCCTGAACGCCCACCACCGCTCTGCAGCCCAGGTATTCCCCGAGCCCCCTGAATCT240
H             ACCTTCCTGAACGCCCACCACCGCTCTGCAGCCCAGGTATTCCCCGAGCCCCCTGAATCT240
MF            ACCTTCCTGAACGCCCACCACCGCTCTGCAGCCCAGGTATTCCCCGAGCCCCCTGAATCT240
              ************************************************************

U77614.1      GACCACGAGGACACAGATTTCGAGCCCTCCCTTCCCGAGTGCCCAGAGTACCAGGAAGAA300
H             GACCACGAGGACACAGATTTCGAGCCCTCCCTTCCCGAGTGCCCAGAGTACCAGGAAGAA300
MF            GACCACGAGGACACAGATTTCGAGCCCTCCCTTCCCGAGTGCCCAGAGTACCAGGAAGAA300
              ************************************************************

U77614.1      GAGTTCGACTACGAATCCGAGACCGAGTCCGAGTCTGAAATCGAGTCCGAGACCGAATTC360
H             GAGTTCGACTACGAATCCGAGACCGAGTCCGAGTCTGAAATCGAGTCCGAGACCGAATTC360
MF            GAGTTCGACTACGAATCCGAGACCGAGTCCGAGTCTGAAATCGAGTCCGAGACCGAATTC360
              ************************************************************

U77614.1      GAGACCGAGTCTGACACCGCCCCCACCACTGAGCCGGAGACCGAGCCCGAGGACGAGCCG420
H             GAGACCGAGTCTGACACCGCCCCCACCACTGAGCCGGAGACCGAGCCCGAGGACGAGCCG420
MF            GAGACCGAGTCTGACACCGCCCCCACCACTGAGCCGGAGACCGAGCCCGAGGACGAGCCG420
              ************************************************************

U77614.1      GGCCCCGTGGTGCCCAAGCGCCCCACCTTCCACCAGTCTCTCACCGAGCGTCTCAGCGCC480
H             GGCCCCGTGGTGCCCAAGCGCCCCACCTTCCACCAGTCTCTCACCGAGCGTCTCAGCGCC480
MF            GGCCCCGCGGTGCCCAAGCGCCCCACCTTCCACCAGTCTCTCACCGAGCGTCTCAGCGCC480
              ******* ****************************************************

U77614.1      CTGAGGCTGCGGAGC      495
H             CTGAGGCTGCGGAGC      495
MF            CTGAGGCTGCGGAGC      495
              ***************
```

Figure S10: The alignment of the NESP55 gene (495 bp) aligns in healthy dairy cows (H) and those with milk fever (MF).

```
NM_001083798.1      ATCGAGCAGAGCATCGAGCAGGAGGAAGGCCTGAACCGCTCCTCCGCAGACCTGAGGATC   60
H                   ATCGAGCAGAGCATCGAGCAGGAGGAAGGCCTGAACCGCTCCTCCGCAGACCTGAGGATC   60
MF                  ATCGAGCAGAGCATCGAGCAGGAGGAAGGCCTGAACCGCTCCTCCGCAGACCTGAGGATC   60
                    ************************************************************

NM_001083798.1      CGTAAGACACAGCACTCCACGCTGTCCCGGAAGTTCGTGGAGGTGATGTCCGAGTACAAC   120
H                   CGTAAGACACAGCACTCCACGCTGTCCCGGAAGTTCGTGGAGGTGATGTCCGAGTACAAC   120
MF                  CGTAAGACACAGCACTCCACGCTGTCCCGGAAGTTCGTGGAGGTGATGTCCGAGTACAAC   120
                    ************************************************************

NM_001083798.1      GCCACGCAGTCTGACTACCGCGAGCGCTGCAAGGGCCGCATCCAGAGGCAGCTGGAGATC   180
H                   GCCACGCAGTCTGACTACCGCGAGCGCTGCAAGGGCCGCATCCAGAGGCAGCTGGAGATC   180
MF                  GCCACGCAGTCTGACTACCGCGAGCGCTGCAAGGGCCGCATCCAGAGGCAGCTGGAGATC   180
                    ************************************************************

NM_001083798.1      ACCGGCCGGACCACGACCAGCGAGGAGCTGGAGGACATGCTGGAGAGTGGGAACCCCGCC   240
H                   ACCGGCCGGACCACGACCAGCGAGGAGCTGGAGGACATGCTGGAGAGTGGGAACCCCGCC   240
MF                  ACCGGCCGGACCACGACCAGCGAGGAGCTGGAGGACATGCTGGAGAGTGGGAACCCCGCC   240
                    ************************************************************

NM_001083798.1      ATCTTTGCCTCCGGGATCATCATGGACTCCAGCATCTCGAAGCAGGCCCTGAGTGAGATT   300
H                   ATCTTTGCCTCCGGGATCATCATGGACTCCAGCATCTCGAAGCAGGCCCTGAGTGAGATT   300
MF                  ATCTTTGCCTCCGGGATCATCATGGACTCCAGCATCTCGAAGCAGGCCCTGAGTGAGATT   300
                    ************************************************************

NM_001083798.1      GAGACACGCCACAGCGAGATCATCAAGCTGGAGAACAGCATCCGGGAGCTGCACGACATG   360
H                   GAGACACGCCACAGCGAGATCATCAAGCTGGAGAACAGCATCCGGGAGCTGCACGACATG   360
MF                  GAGACACGCCACAGCGAGATCATCAAGCTGGAGAACAGCATCCGGGAGCTGCACGACATG   360
                    ************************************************************

NM_001083798.1      TTCATGGACATGGCCATGCTCGTGGAGAGCCAGGGGGAGATGATTGACAGGATTGAGTAC   420
H                   TTCATGGACATGGCCATGCTCGTGGAGAGCCAGGGGGAGATGATTGACAGGATTGAGTAC   420
MF                  TTCATGGACATGGCCATGCTCGTGGAGAGCCAGGGGGAGATGATTGACAGGATTGAGTAC   420
                    ************************************************************

NM_001083798.1      AACGTGGAACATTCGGTGGACTACGTGGAGAGGGCCGTGTCTGACACCAAGAAGGCCG     478
H                   AACGTGGAACATTCGGTGGACTATGTGGAGAGGGCCGTGTCTGACACCAAGAAGGCCG     478
MF                  AACGTGGAACATTCGGTGGACTACGTGGAGAGGGCCGTGTCTGACACCAAGAAGGCCG     478
                    *********************** **********************************
```

Figure S11: The alignment of the STX1A gene (478 bp) aligns in healthy dairy cows (H) and those with milk fever (MF).

```
NM_001427871.1      ACTCAGGATCCGCTGTATGGTTACTTTGCTGCTGTAGCTGGACAGGATGGACAAATAGAT   60
H                   ACTCAGGATCCGCTGTATGGTTACTTTGCTGCTGTAGCTGGACAGGATGGACAAATAGAT   60
MF                  ACTCAGGATCCGCTGTATGGTTACTTTGCTGCTGTAGCTGGACAGGATGGACAAATAGAT   60
                    ************************************************************

NM_001427871.1      GCTGATGAACTGCAAAGATGCCTGACACAGTCGGGCATTGCTGGAGGATACAAACCTTTT   120
H                   GCTGATGAACTGCAAAGATGCCTGACACAGTCGGGCATTGCTGGAGGATACAAACCTTTT   120
MF                  GCTGATGAACTGCAAAGATGCCTGACACAGTCGGGCATTGCTGGAGGATACAAACCTTTT   120
                    ************************************************************

NM_001427871.1      AACCTGGAGACTTGCCGGCTTATGGTTTCAATGCTGGATAGAGACATGTCAGGCACAATG   180
H                   AACCTGGAGACTTGCCGGCTTATGGTTTCAATGCTGGATAGAGACATGTCAGGCACAATG   180
MF                  AACCTGGAGACTTGCCGGCTTATGGTTTCAATGCTGGATAGAGACATGTCAGGCACAATG   180
                    ************************************************************

NM_001427871.1      GGTTTCAATGAATTTAAAGAACTCTGGGCTGTACTGAATGGCTGGAGACAACACTTTATC   240
H                   GGTTTCAATGAATTTAAAGAACTCTGGGCTGTACTGAATGGCTGGAGACAACACTTTATC   240
MF                  GGTTTCAATGAATTTAAAGAACTCTGGGCTGTACTGAATGGCTGGAGACAACACTTTATC   240
                    ************************************************************

NM_001427871.1      AGTTTCGACAGTGATAGGAGTGGAACAGTGGATCCCCAAGAATTGCAGAAGGCCCTGACA   300
H                   AGTTTCGACAGTGATAGGAGTGGAACAGTGGATCCCCAAGAATTGCAGAAGGCCCTGACA   300
MF                  AGTTTCGACAGTGATAGGAGTGGAACAGTGGATCCCCAAGAATTGCAGAAGGCCCTGACA   300
                    ************************************************************

NM_001427871.1      ACAATGGGATTTAGGTTGAGTCCCCAGGCTGTGAATTCAATTGCAAAACGATACAGTACC   360
H                   ACAATGGGATTTAGGTTGAGTCCCCAGGCTGTGAATTCAATTGCAAAACGATACAGTACC   360
MF                  ACAATGGGATTTAGGTTGAGTCCCCAGGCTGTGAATTCAATTGCAAAACGATACAGTACC   360
                    ************************************************************

NM_001427871.1      AATGGAAAGATCACCTTCGATGATTACATCGCCTGTTGCGTCAAGCTGCGAGCTCTAACA   420
H                   AATGGAAAGATCACCTTCGATGATTACATCGCCTGTTGTGTCAAGCTGCGAGCTCTAACA   420
MF                  AATGGAAAGATCACCTTCGATGATTACATCGCCTGTTGCGTCAAGCTGCGAGCTCTAACA   420
                    ************************************** *********************

NM_001427871.1      GACAGCTTTCGAAGACGGGATACTGCTCAGCAAGGTGTAGTAAATTTCCCATATGATGAT   480
H                   GACAGCTTTCGAAGACGGGATACTGCTCAGCAAGGTGTAGTGAATTTCCCATATGATGAT   480
MF                  GACAGCTTTCGAAGACGGGATACTGCTCAGCAAGGTGTAGTAAATTTCCCATATGATGAT   480
                    ***************************************** ******************

NM_001427871.1      TTCATCCAGTGTGTCATGAGTG    502
H                   TTCATCCAGTGTGTCATGAGTG    502
MF                  TTCATCCAGTGTGTCATGAGTG    502
                    **********************
```

Figure S12: The alignment of the SRI gene (502 bp) aligns in healthy dairy cows (H) and those with milk fever (MF).

```
NM_001435139.1      TTAGCAGCAGAGGTAGACCCTGACTTTGAGGAAGAATGCCTGGAGTTTCAGCCCTCAGTG   60
H                   TTAGCAGCAGAGGTAGACCCTGACTTTGAGGAAGAATGCCTGGAGTTTCAGCCCTCAGTG   60
MF                  TTAGCAGCAGAGGTAGACCCTGACTTTGAGGAAGAATGCCTGGAGTTTCAGCCCTCAGTG   60
                    ************************************************************

NM_001435139.1      GACCCTGATCAGGATGCCTCCCGAAGCAGGTTGCGAAATGCCCAAGAGAAGATGGTGTAC   120
H                   GACCCTGATCAGGATGCCTCCCGAAGCAGGTTGCGAAATGCCCAAGAGAAGATGGTGTAC   120
MF                  GACCCTGATCAGGATGCCTCCCGAAGCAGGTTGCGAAATGCCCAAGAGAAGATGGTGTAC   120
                    ************************************************************

NM_001435139.1      TCCCTGGTCTCTGTGCCCGAGGGCAATGACATCTCCTCCATTTTTGAGCTGGACCCCACG   180
H                   TCCCTGGTCTCTGTGCCCGAGGGCAATGACATCTCCTCCATTTTTGAGCTGGACCCCACG   180
MF                  TCCCTGGTCTCTGTGCCCGAGGGCAATGACATCTCCTCCATTTTTGAGCTGGACCCCACG   180
                    ************************************************************

NM_001435139.1      ACGCTCCGTGGAGGCGACAGCCTCGTCCCGAGAAACTCCTATGTCCGGCTCCGACACCTG   240
H                   ACGCTCCGTGGAGGCGACAGCCTCGTCCCGAGAAACTCCTATGTCCGGCTCCGACACCTG   240
MF                  ACACTCCGTGGAGGCGACAGCCTCGTCCCGAGAAACTCCTATGTCCGGCTCCGACACCTG   240
                    ** *********************************************************

NM_001435139.1      TGTACAAATACCTGGGTTCACAGCACAAACATCCCCATTGACAAGGAAGAAGAAAAACCC   300
H                   TGTACAAATACCTGGGTTCACAGCACAAACATCCCCATTGACAAGGAAGAAGAAAAACCC   300
MF                  TGTACAAATACCTGGGTTCACAGCACAAACATCCCCATTGACAAGGAAGAAGAAAAACCC   300
                    ************************************************************

NM_001435139.1      GTGATGCTGAAAATTGGCACTTCTCCCGTGAAGGAGGACAAGGAAGCCTTTGCCATAGTG   360
H                   GTGATGCTGAAAATTGGCACTTCTCCCGTGAAGGAGGACAAGGAAGCCTTTGCCATAGTG   360
MF                  GTGATGCTGAAAATTGGCACTTCTCCCGTGAAGGAGGACAAGGAAGCCTTTGCCATAGTG   360
                    ************************************************************

NM_001435139.1      CCAGTCTCTCCTGCTGAG        378
H                   CCAGTCTCTCCTGCTGAG        378
MF                  CCAGTCTCTCCTGCTGAG        378
                    ******************
```

Figure S13: The alignment of the ITPR1gene (378 bp) aligns in healthy dairy cows (H) and those with milk fever (MF).

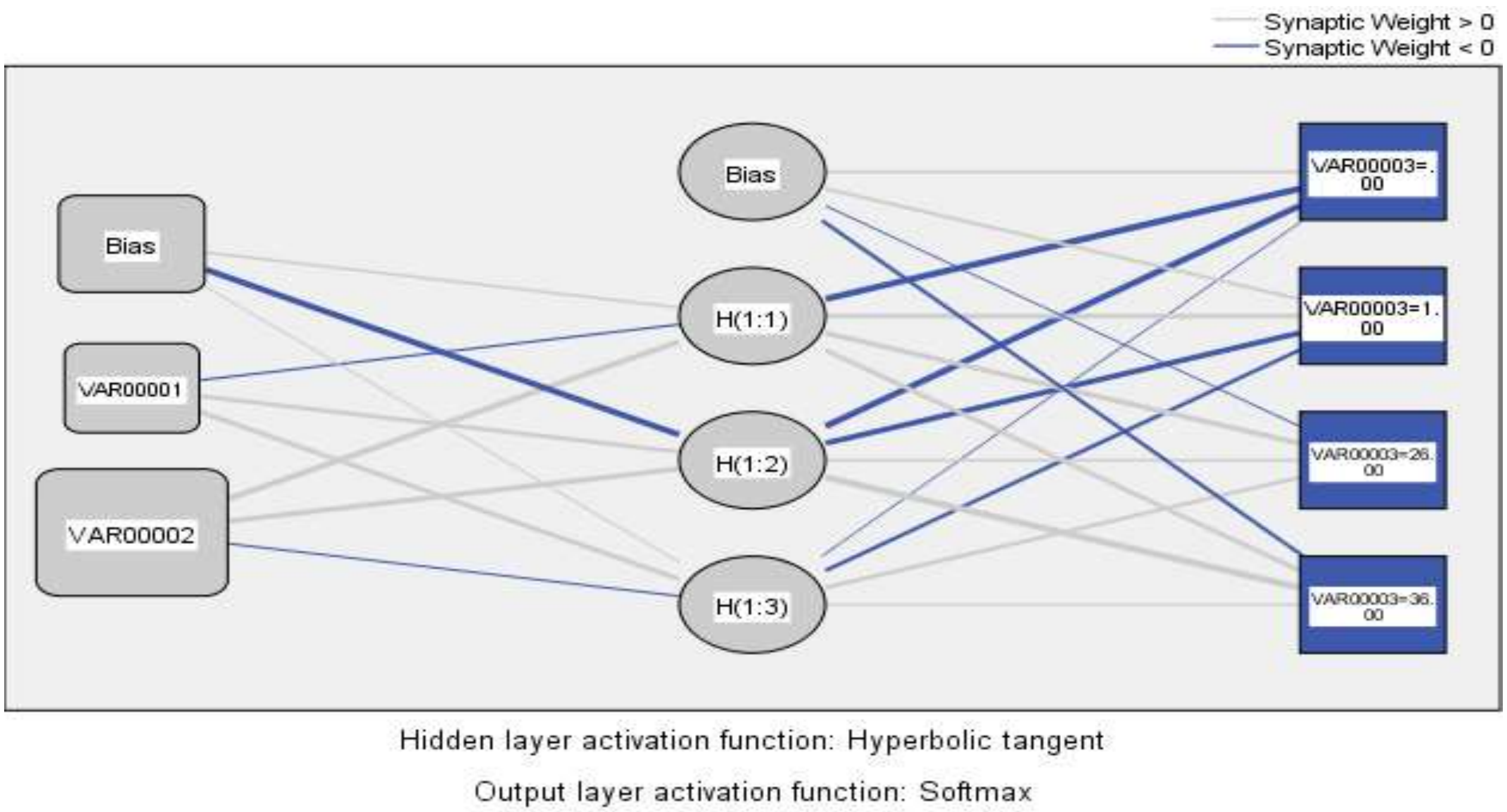

Figure S14. Relative importance of candidate transcriptomic biomarkers for milk fever prediction obtained from the artificial neural network model. Biomarkers are ranked according to normalized importance, with higher values indicating a greater contribution to disease classification.
